# Prevalence and Characteristics of *Staphylococcus aureus* Associated with Meat and Meat Products in African Countries: A Review

**DOI:** 10.3390/antibiotics10091108

**Published:** 2021-09-14

**Authors:** Thembeka Thwala, Evelyn Madoroba, Albert Basson, Patrick Butaye

**Affiliations:** 1 Department of Biochemistry and Microbiology, University of Zululand, Private Bag X1001, KwaDlangezwa 3886, South Africa; mmbeka@gmail.com (T.T.); MadorobaE@unizulu.ac.za (E.M.); BassonA@unizulu.ac.za (A.B.); 2 Department of Biosciences, Ross University School of Veterinary Medicine, West Farm, Saint Kitts and Nevis; 3 Bacteriology and Avian Diseases, Department of Pathology, Faculty of Veterinary Medicine, Ghent University, 9820 Merelbeke, Belgium

**Keywords:** *Staphylococcus aureus*, Africa, antimicrobial resistance, livestock-associated methicillin-resistant *Staphylococcus aureus*, meat and meat products

## Abstract

Antimicrobial resistance has been increasing globally, which negatively affects food safety, veterinary, and human medicine. Ineffective antibiotics may cause treatment failure, which results in prolonged hospitalisation, increased mortality, and consequently, increased health care costs. *Staphylococcus aureus* causes a diverse range of infections including septicaemia and endocarditis. However, in food, it mainly causes food poisoning by the production of enterotoxins. With the discovery of methicillin-resistant *S. aureus* strains that have a separate reservoir in livestock animals, which were termed as livestock-associated methicillin-resistant *S. aureus* (LA-MRSA) in 2005, it became clear that animals may pose another health risk. Though LA-MRSA is mainly transferred by direct contact, food transmission cannot be excluded. While the current strains are not very pathogenic, mitigation is advisable, as they may acquire new virulence genes, becoming more pathogenic, and may transfer their resistance genes. Control of LA-MRSA poses significant problems, and only Norway has an active mitigation strategy. There is limited information about LA-MRSA, MRSA in general, and other *S. aureus* infections from African countries. In this review, we discuss the prevalence and characteristics of antimicrobial susceptible and resistant *S. aureus* (with a focus on MRSA) from meat and meat products in African countries and compare it to the situation in the rest of the world.

## 1. Introduction

The increase in antimicrobial-resistant zoonotic bacterial pathogens has become a significant public health challenge. There are diverse drivers of antimicrobial resistance, with the use of antimicrobial agents in food-producing animals as a major driver of resistance in animal bacteria [1]. Ineffective antimicrobials may cause treatment failure, which results in prolonged hospitalisation and increased mortality, and consequently, increased health care costs.

*Staphylococcus aureus* causes a diverse range of infections in humans. These include severe diseases such as septicaemia and endocarditis, which are frequently associated with high mortality if not treated properly [2]. This bacterium has been reported in the last few decades to be resistant to many of the available antimicrobial agents, and recently, it became resistant to one of the last lines of treatment daptomycin and linezolid [3,4]. However, the most common cause of nosocomial and community-associated staphylococcal infections is methicillin-resistant *S. aureus* (MRSA) [5].

*S. aureus* has been documented as one of the most significant causes of hospital-acquired infections in the past decades. It belongs to the ESKAPE group of bacteria (*Enterococcus* spp., *S. aureus*, *Klebsiella* spp., *Acinetobacter baumannii*, *Pseudomonas aeruginosa*, *Enterobacter* spp.), all of which have multi-drug resistant profiles [6]. *S. aureus* has been associated with a high rate of antimicrobial resistance in both hospitals and other environments such as community settings [7]. While there was a slight decrease in infections with MRSA in the US, Europe, Canada, and South Africa, in some regions such as sub-Saharan Africa, the trends increased [8]. This increase is a public health concern.

*S. aureus* acquired resistance to methicillin and most other beta-lactamase resistant beta-lactam antibiotics through the acquisition of the *mec* gene located on the staphylococcal cassette chromosome *mec* (SCC*mec*) element [5]. While the *mecA* gene is the most prevalent, *mecC* has been mainly associated with animals, and it has also been detected in human and environmental staphylococci [9]. The MRSA was first described in 1961, and this occurred soon after methicillin was used as the first line of treatment for penicillin-resistant *S. aureus* [10]. Since then, MRSA has been reported worldwide in hospitals and from the early 1990s in community settings. During the early 1990s, oxacillin, and later on cefoxitin, was preferred over methicillin for testing the staphylococci, but the abbreviation MRSA is still used for historical reasons [11]. The promoter of the *mecA* genes can have several mutations, which may contribute to the variation of oxacillin MICs [12]. Some *S. aureus* strains may even be susceptible to oxacillin in vitro while simultaneously harbouring *mecA*, and they are referred to as OS-MRSAs [13]. OS-MRSAs have been associated with food, animals, and clinical strains and are important as causative agents of clinical challenges, as they may acquire resistance to β-lactams effortlessly [13]. Livestock-associated methicillin-resistant *S. aureus* (LA-MRSA) was only detected in 2005 and was found mainly amongst pigs and veal calves [9].

Contamination of food by *S. aureus* results from poor hygiene practices during food processing and storage. Both human- and animal-associated strains can be involved [14]. However, the strains that produce staphylococcal enterotoxins and mainly the staphylococcal enterotoxin A (SEA), encoded by the *sea* gene, cause food poisoning outbreaks [15]. The other toxins that may be involved are encoded by the *seb*, *sec*, *sed* and *see* enterotoxin genes [16].

Food poisoning by the staphylococcal toxin is characterised by diarrhoea, nausea, abdominal cramping, and vomiting within 24 h of ingestion [17]. However, with the advent of LA-MRSA, other health risks than food poisoning should be considered. Different studies have indicated that LA-MRSA (and other types of MRSA) can be present in milk, poultry, cattle, pigs, and pets [14,17,18,19,20,21]. In Europe, the most prevalent LA-MRSA clonal complex is CC398; however, other lineages such as ST1, ST5, ST9, ST97, ST130, and ST433 have been reported. In Asia, the majority of the strains belong to CC9, while in North America, CC398 and CC9 were equally common [21]. Though LA-MRSA is mainly transferred by direct contact, food transmission cannot be excluded. While the current strains are not very pathogenic, mitigation strategies are advisable, as these bacteria may acquire new virulence genes and become more pathogenic [9]. Moreover, the transfer of resistance genes should also be taken into account.

There is limited information about MRSA, LA-MRSA, and other *S. aureus* infections from African countries. In this review, we discuss the prevalence and characteristics of *S. aureus*, including MRSA recovered from meat and meat products in African countries, and evaluate the extent of antimicrobial resistance of *S. aureus* recovered from humans and animals in African countries, compared to other regions.

## 2. The Prevalence of *S. aureus* in Meat and Meat Products in African Countries

Animals such as cattle, pigs, chickens, turkey, horses, sheep, and man can be colonised by *S. aureus* on their skin and in their nares [22]. Typically, different clones are associated with specific hosts, though some clones lack host specificity while others can occasionally infect other species [23]. Few studies in Africa have described *S. aureus* in meat and meat products. Moreover, few isolates have been characterized in detail. Different types of meat may have a different prevalence and, in this section, we handle the different meat types separately.

### 2.1. Prevalence of S. aureus in Raw Unprocessed Red Meat

In Table 1, a summary of the studies performed on raw unprocessed red meat in Africa is listed.

We could find data on a total of 2853 samples from all over Africa. Overall, 699 were contaminated with *S. aureus*. Positive samples included raw unprocessed beef, pork, goat meat, camel meat, lamb/sheep, and unspecified red meat products. The total prevalence of *S. aureus* in meat products was 24.5%, and of all red meats, beef samples showed the highest prevalence with 33.08% of the samples being positive. These findings indicate a higher potential risk of beef for human infections. Compared to data from other countries, the results are similar [36,37,38,39], though in China, a higher percentage of contamination was found, with a little over 50% of the raw beef samples found positive [40], as well as in a specific study from the US where a prevalence of 65.6% was found in retail beef [14], while in another US study, the overall positivity *S. aureus* was 16.4% [41]. This large variation in both Africa and worldwide may be attributed to several factors, including sampling method, isolation methods, sampling site on the carcass, different cuts of meat, contamination during and after the slaughter, storage of the meat, as well as the processing of the meat [42]. A total of 691 pork samples were analysed in Africa (Table 1), and almost 14% of them were found positive for *S. aureus*. These findings are similar to what was found in the US [38], while much higher percentages were found in other studies from the US and Brazil [39,43,44,45]. In Asia, a lower prevalence was found [40].

There was quite some variation in the prevalence between the African countries, even between studies from the same country as exemplified by the Nigerian studies. In Nigeria, there was an apparent increase in prevalence [27,46], and the reasons for this apparent increase remain unclear. In South Africa, two studies performed in the same year showed also substantial differences [25,26]. However, the studies used a different type of sample and a different way of collecting the sample, different isolation methods, and were conducted in a different geographical location. In Ivory Coast, similar to beef, the prevalence was lowest [24]. Additionally, here it is clear that the few data available do not allow a broad conclusion on the prevalence nor evolution of the contamination of meat products in Africa. Hence, it is necessary to conduct more research on the subject in Africa.

Only one study reported the prevalence of *S. aureus* from goat meat [27]. The prevalence was on the lower side. Similarly, only two studies were conducted on the prevalence of *S. aureus* in sheep meat. Both were conducted in South Africa, and the results showed major differences [25,26]. Only Egypt reported the prevalence of *S. aureus* from camel meat [32]. Those studies do not allow to have a good view on the prevalence of *S. aureus* in those meats; however, it indicates that also those meat products can substantially be contaminated. Surprisingly, on unspecified raw red meat in Nigeria, nearly all products were contaminated [34], while a former study showed a low level of contamination [33].

The distribution of *Staphylococcus aureus* in red meat differs, thus widely based on different studies, and it seems to be dependent on both the country and the meat product, though other factors cannot be excluded. No firm conclusion on differences between countries, in time or red meat type, can be made, as the studies are too limited and too different. It is, however, clear that *S. aureus* contamination of red meat products is, and remains, a problem that needs mitigation.

### 2.2. Prevalence of S. aureus in Raw Unprocessed Poultry

Compared to red meats, quite a lot more samples from poultry have been analysed, though the numbers are still limited relative to the vastness of the African continent. Moreover, the studies have been performed in a limited number of counties (Table 2). We could find data from 11,080 samples of chicken meat, and of those, a little over 20% were found to be contaminated with *S. aureus* (Table 2). The prevalence of *S. aureus* in poultry meat seems to be lower than in beef. The oldest study is from Nigeria and was performed in 2009 on a relatively small number of samples. The prevalence was very high, with 80% of the samples being positive [27]. A second larger study performed in 2011 from Tunisia showed, however, only 51.19% of the samples were positive [47]. Due to differences in sample size, it is difficult to assess whether there was a potential evolution. Similarly, in South Africa, the prevalence seems to drop over time, but it remained a high prevalence [25,26]. Other studies in African countries were limited, though one very large study in Algeria showed a prevalence of close to 20%, which is close to the average prevalence measured for all of Africa [28].

The overall prevalence found in Africa is similar to what has been found in China and Brazil [45,48]. In the US, a higher prevalence was found in most of the studies, though one study showed a prevalence as low as 0.3% [39,43,49]. There are few data available from Asiatic countries, and the prevalence rates were overall lower than in most other countries [48,50]; however, a conflicting study reported a higher prevalence of 70% [20]. From the data published, it is clear that poultry in Africa is also contaminated with *S. aureus*, though to a lesser extent than most other studies.

### 2.3. Prevalence of S. aureus in Raw Processed Meat

Table 3 shows the studies that have determined the prevalence of *S. aureus* in raw processed meats. All were beef samples. Out of 247 tested samples, 31 (13%) were positive for *S. aureus*. One would expect higher levels of contamination, compared to unprocessed meat, as processed meat is more prone to microbial contamination. However, we could not confirm this with the published data. It should be noted also that some processes such as smoking and drying may reduce contamination, and from the above studies, the meat used was dried and smoked. As no ample data are present, no real conclusions can be drawn for this type of food.

### 2.4. Prevalence of S. aureus in Ready-to-Eat Meat

Table 4 indicates the prevalence of *S. aureus* from ready-to-eat meat, and only one study was found and few samples were included. This study showed a low prevalence of *S. aureus*.

The contamination of meat by *S. aureus* across the food chain is a complicated process. The contamination may originate from animals, as well as from humans. It has been shown before that in humans, the main source of contamination is food handlers that carry *S. aureus* in their noses or hands [54]. Improper hygiene at that level should be avoided to reduce the odds of meat contamination and food poisoning. However, due to the multitude of small slaughter and meat processing operations in Africa, this may prove to be difficult.

The other source of contamination is the animals themselves. Food-producing animals carry *S. aureus* on their skin, nose, as well as in their intestine. The main factors that influence the level of contamination are the length at which animals are transported and the methods which are used to move animals from one place to another, holding conditions, geographic location, as well as climate changes [55]. Next to that, it is always important to follow proper slaughter and food handling protocols to minimise contamination with pathogenic microorganisms. The growth of *S. aureus* and the production of enterotoxins in food is caused by improper handling of foods and the improper storage conditions that support the growth of this pathogen [56]. In developing countries, pathogens are being transmitted from livestock to humans through contact or contaminated meat or meat products [57]. Only typing of the strains can bring clarity in the origin of the contamination and the public health risk associated with the contamination.

In Africa, red meat has the highest prevalence of *S. aureus*, compared to other types of meat. *S. aureus* has been reported to be one of the pathogens causing foodborne infections from different regions worldwide. Findings indicate a specific problem on the African continent in beef production that needs further investigation.

Few studies in Africa have assessed contamination sites across the food chain. However, in Ethiopia, *S. aureus* was found on all equipment that was used in the abattoirs [58].

From the results obtained in our review, more research needs to be conducted in Africa to fully understand the prevalence and way of spread of *S. aureus* via the food chain. It is also important that African countries should improve the hygiene conditions, especially in abattoirs, butcheries, and retail shops to improve public health. *S. aureus* has been associated with foodborne diseases in different parts of the world, and infections caused by this pathogen are difficult to treat because some strains are resistant to antibiotics.

## 3. Antimicrobial Resistance in *S. aureus* from Meat and Meat Products in Africa

The data for antimicrobial resistance in different types of meat from various African countries comprised 20 antibiotics that were tested, representing 10 different classes of antibiotics (Table 5). Not all studies used the same antibiotics, hence the difference in the number of strains tested. Studies originated from Nigeria, South Africa, Cameroon, Algeria, Tunisia, and Egypt, and the cumulated results are represented in Table 5. Due to the limited number of studies and the limited number of strains involved, few conclusions can be drawn on differences between regions. Nevertheless, an overall view may help in what antimicrobial resistance is present in Africa. Due to the differences in the numbers of strains tested, the resistance percentages within an antibiotic class may vary. Therefore, the data presented only give an impression of the resistance problem.

High resistance was observed from ampicillin (50%), clindamycin (33.8%), doxycycline (27%), and ofloxacin (57%), though ofloxacin represented a small sample size.

Upon examination of the data in more detail, we observe that the resistance against beta-lactam antibiotics is in general quite high. It is surprising to see such high resistance against the penicillinase-resistant β-lactam antibiotics, as the isolation methods that were used were not specifically targeting MRSA. It should be noted, however, that in the concerned studies, the resistance was not confirmed by PCR and may thus be lower [28,32,34,54,59]. Nevertheless, it is an indication that the levels of MRSA on meat samples may be quite high. It has indeed been shown that meat can be contaminated by LA-MRSA, especially pork, as the prevalence of LA-MRSA is high in pigs, as well as by human MRSA strains [60]. Nevertheless, MRSA should be confirmed by PCR, as the disk diffusion test is not very accurate for the determination of MRSA [61]. Thus, the true prevalence of MRSA in meat remains obscure. Interestingly, one group tested for ceftaroline, an anti-MRSA antibiotic, and the resistance is high [26]. Vancomycin (0.7%) and gentamicin (1%) showed very low resistance, and only against amikacin, there was quite some resistance. Resistance against vancomycin in *S. aureus*, as determined by the disk diffusion test, is not very reliable [62], and the presence of vancomycin resistance in *S. aureus* may thus be questionable. Resistance against the tetracyclines is somewhat varying between studies, as was the case with the difference in the prevalence of resistance against the different antibiotics in this class. A similar observation can be made for fluoroquinolones. Resistance against other antibiotics was medium to low. Apart from the presence of MRSA, there does not seem to be a very large problem with resistance in this bacterium on meat and meat products in Africa.

Compared to other parts in the world where antimicrobial resistance has been assessed on strains from food, similar resistance percentages were noted such as in China, [63,64], the USA [39], Greece [65], India [66], Italy [37,67,68]. Few other studies showed differences, including a German study, which showed high resistance in tetracycline, oxacillin, erythromycin, clindamycin, kanamycin, gentamicin, and ciprofloxacin in *S. aureus* from turkey meat [69].

## 4. Antimicrobial Resistance Genes Detected in *S. aureus* from Meat in Africa

Only two studies reported the resistance genes present in *S. aureus* (MSSA) from meat products in Africa (Table 6). The genes identified are commonly identified worldwide [76]. However, since the data come only from two countries, with each one being a limited study (Table 6), conclusions are difficult to make.

## 5. Genotyping of Different *S. aureus* Meat Isolates from Africa

The genetic background of only a few strains has been determined, and all came from a single study in Tunisia (Table 7) [47]. Four strains were MSSA ST398, which is surprising as most ST398 strains are MRSA and came from chicken and veal. MSSA CC398 has been mainly associated with humans [77], where the human evasion cluster genes IEC are, in general, present [78]. Other clonal complexes detected included ST22 which is also mainly associated with human infections [79], though it has also been associated with infections in animals including outbreaks in veterinary clinics [80,81,82]. The ST8 clone is a typical community-acquired clone also associated with hospital outbreaks, usually being an MRSA as the USA300 clone [83], which has been reported worldwide, including sub-Saharan Africa [84,85]. The presence of MSSA ST398, ST8, and ST22 indicates that they might be more associated with humans contaminating the meat, either during slaughter, processing, or storage.

## 6. MRSA in Meat Products in Africa

### 6.1. Prevalence and Characteristics of Methicillin-Resistant S. aureus (MRSA) in Different Types of Meat

We separated the studies dealing with the detection of MRSA from the other studies, as they applied a very different methodology. Using selective isolation, the studies were focusing only on the presence of a specific subtype of *S. aureus* and thus not giving a full picture of the presence of *S. aureus*. Moreover, these MRSA have never been implicated in food poisoning and have thus a more zoonotic aspect. These strains can be of animal origin (the LA-MRSA) or they can originate from humans. The LA-MRSA are specific types of MRSA that can be distinguished by molecular typing. Therefore, the prevalence of LA-MRSA is mainly related to carcass contamination from animal origin, rather than other origins. Therefore, the prevalence of LA-MRSA is in direct relation with the prevalence in the animal itself, although not excluding cross-contamination during slaughter and processing.

We found data on the selective isolation of MRSA from a total of 3746 meat samples which included beef, pork, and poultry (Table 8). The numbers should be interpreted with care, however, as not all MRSA cases were confirmed, and false-positive results are possible. This is also evident in Table 6, where not all strains are resistant to all β-lactam antibiotics. The highest prevalence of MRSA was observed in pork (12%), which is the animal species with the highest prevalence of LA-MRSA worldwide. However, the carriage differs across the countries, and the prevalence at pig meat level is, in general, lower than the prevalence at farm level [86]. The prevalence rates in poultry and beef were 6.76% and 6.12%, respectively. The prevalence of LA-MRSA in these latter species has been shown to be lower than in pigs except for veal calves [86]. The prevalence of MRSA in pork in Africa is, however, much lower, compared to what has been found in countries with a high prevalence of LA-MRSA in pigs [45,67,87,88,89]. More research is required in order to determine whether the prevalence of LA-MRSA in animals is indeed lower in Africa. Nevertheless, the prevalence of MRSA in pork from countries such as China and the USA, where the prevalence in pigs is moderate to high, was lower [36,41,43,64]. The prevalence of MRSA in chicken meat from Africa (6.76%) was lower or similar, compared to most other studies [20,41,45,48,64,90,91].

Prevalence in beef was 6.12%, which was higher than 4%, 0.8%, and 1.7% found in beef meat from the USA [36,38,49] and also 4.4% found in Hong Kong [91], while in Brazil, a very high prevalence (23.3%) was found [45]. However, more research is required in order to ascertain the extent of MRSA contamination of meat in Africa.

### 6.2. Antimicrobial Resistance Profile of MRSA in African Countries

From Table 9, it is clear that not all tests were performed accurately, as MRSA is resistant to all beta-lactam antibiotics that were included in testing (there were no anti-MRSA β-lactams included). Nevertheless, several strains were not confirmed by PCR to be MRSA, which might affect the final results. Resistance was commonly found against tetracyclines, which is indicative of the presence of LA-MRSA ST398. These strains are classically nearly 100% resistant to tetracyclines. LA-MRSA is also typically multidrug resistant, with high resistance percentages against aminoglycosides, macrolides, and lincosamides, as well as fluoroquinolones, though this may vary a bit according to the studies [88,94,95,96,97]. Thus, the data strongly indicate that many MRSA could be LA-MRSA. The high prevalence of vancomycin resistance has to be interpreted with care, as the studies were carried out using the disk diffusion test, and this is not very reliable [98,99]. Vancomycin resistance has never been reported before in LA-MRSA and is extremely rare in other MRSA.

Unfortunately, there are little data from Africa on the prevalence of LA-MRSA [42], though it is present in animals. It has indeed been shown that meat can be contaminated by LA-MRSA, especially pork since the prevalence of LA-MRSA is, in general, highest in pigs [60]. Nevertheless, MRSA should be confirmed by PCR, as the disk diffusion test is not very accurate for the determination of MRSA [61]. The true prevalence of MRSA in meat remains thus unclear.

## 7. Antimicrobial Resistance Genes Detected in MRSA from Meat in Africa

In few studies, a selection of resistance genes in MRSA have been determined, which makes the data rather incomplete (Table 10). The genes found in Africa are the classical genes found worldwide, though one big exception is the presence of *vanA* and *vanB* in Egyptian camel and beef isolates [30,32]. Vancomycin resistance mediated by the *vanA* or *vanB* gene in staphylococci has rarely been described in staphylococci and is mainly associated with vancomycin resistance in enterococci [10]. The reason for this presence of vancomycin could be the use of avoparcin, another glycopeptide antibiotic, which is frequently used in Egypt for the prevention of necrotic enteritis and as a growth promoter in the poultry industry [100]. Another description of vancomycin resistance in Egypt was from ready-to-eat food (beef burgers and hot dogs); however, in these four strains, no *van* gene-mediated resistance could be detected [101]. Unfortunately, these strains have not been typed, and therefore, it is unclear whether these might be strains of human or animal origin. A survey conducted from Egypt hospital on dairy food, food handlers, and patients indicated the presence of methicillin-resistant, coagulase-positive, and vancomycin-resistant staphylococci but only from humans (food handlers and patients) [102], whereas coagulase-negative staphylococci (both MR and VR) were identified from food, food handlers, and patients; however, antimicrobial resistance genes were not studied [102]. Hospital infections with VRSA have been described on occasions in Egypt [103]. The presence of vancomycin resistance in MRSA is highly concerning, as it renders an infection with these bacteria very hard to treat. The situation seems unique to Egypt.

## 8. Genotyping of MRSA Meat Isolates from Africa

In South Africa, ST612 (a CC8 strain) has been associated with human infections and has been identified as a dominant clone in hospitals in Cape Town and other provinces [71,104]. However, this clone has also been frequently isolated from animals in other continents such as horses in Australia [105], leading to the detection of the strains also in veterinarians working with horses and other people in contact with horses [67,106,107,108], which indicates the possible transmission from humans to animals. This clone was also multidrug resistant, hence the importance of its spread through the food chain [109].

The presence of ST398 (CC398) indicates that the situation in North Africa might be more similar to Europe, as this is the most detected LA-MRSA clone in animals. Probably this is due to its closer geographical location. The ST398 clonal complex is associated with multi-resistance, including heavy metal resistance, which highlights the need for more studies in Africa on its prevalence. There is thus a need for more research on the prevalence and characteristics of LA-MRSA in African countries [29].

The current data are not representative of what types of MRSA can be found in meat from Africa, nor even of the two countries on which we have information regarding typed strains. The typed MRSA isolates are derived from a single study in Tunisia and two studies from South Africa (Table 11) [47,71,110]. Only in the study by Chairat, typical LA-MRSA ST398 were detected, next to an ST30 strain, which is known to be human associated [47]. In South Africa, one ST239 was found, which is peculiar, as ST239 has been found in animals only on rare occasions and only in Belgium [111,112,113]. ST239 has been mainly reported as an MRSA clone spreading in Asia, though it has also been detected in South Africa and other African countries [114,115,116,117]. It is thus not clear whether this strain might be of animal or human origin, as there are not enough data on the types of MRSA in animals in South Africa.

## 9. Conclusions

Unfortunately, there is little information on *S. aureus* from meat products in Africa, and there is even less on the types and their antimicrobial resistance. Nevertheless, the observation is that meats can be heavily contaminated with *S. aureus*, including MRSA, and this represents a potential public health threat. There is thus an urgent need for more studies on the subject to be able to estimate the public health burden. The current studies are fragmentary using different methodologies, which makes it difficult to compare studies. While the MRSA clones on meat detected in few studies seem to be mainly of human origin, the presence of LA-MRSA CC398 warrants further investigation, as does the presence of VISA.

## Figures and Tables

**Table 1:** Prevalence of *S. aureus* (non-MRSA) in raw unprocessed red meat in African countries.

Meat Type	Year	Country	No. of Tested Samples	No. of *S. aureus positive*	Percentage Prevalence (CI) *	Reference
Beef	2010	Ivory coast	80	12	15 (8–25)	[24]
	2018	South Africa	188	48	25 (19.5–32)	[25]
	2015–2016	South Africa	500	101	20 (16.8–24)	[26]
	2009	Nigeria	75	21	28 (18.2–40)	[27]
	2009–2014	Algeria	465	255	55 (50.2–59)	[28]
	2015	Egypt	27	9	33 (16.5–54)	[29]
	2018	Egypt	25	4	16 (4.5–36)	[30]
Total			1360	450	33.08 (30.6–36)	
Pork	2009	Nigeria	75	9	12 (5.6–22)	[27]
	2018	South Africa	200	14	7 (3.9–11)	[26]
	2018	South Africa	36	8	22 (10.1–39)	[25]
	2010	Ivory Coast	80	5	6.25 (2.1–14)	[24]
	2015	Canary island	300	58	19 (15–24)	[31]
Total			691	94	13.6 (11.1–16)	
Goat meat	2009	Nigeria	75	9	12 (5.6–22)	[27]
Camel meat	2017	Egypt	200	29	14.5 (9.9–20)	[32]
Sheep/lamb		South Africa	300	10	3.3 (1.6–6)	[26]
		South Africa	86	46	53.48 (42.4–64)	[25]
Total			386	56	14.5 (11.1–18)	
Unspecified raw red meat		Nigeria	153	3	9.15 (0.4–6)	[33]
		Nigeria	60	58	98.33 (88.5–100)	[34]
Total			213	61	28.6 (22.7–35)	
Overall total			2853	699	24.5 (22.9–26)	

* Confidence interval: the confidence interval (CI) was set at 95% and calculated based on the exact binomial method [35].

**Table 2:** Prevalence of *S. aureus* in raw unprocessed poultry in African countries.

Meat Type	Year	Country	No. Tested Samples	No. *S. aureus* Positive	Prevalence % (CI)	Reference
Turkey	2012–2013	Algeria	70	32	45 (33.7–58)	[51]
Chicken	2009	Nigeria	75	60	80 (69.2–88)	[27]
	2010–2011	Tunisia	84	41	51.19 (37.7–60)	[47]
	2009–2014	Algeria	8375	1567	18.71 (17.9–20)	[28]
	2015	Egypt	50	3	6 (1.3–17)	[29]
	2015–2016	South Africa	311	106	34.1 (28.8–40)	[52]
	2017	Nigeria	1800	232	13 (11.4–15)	[53]
	2012–2013	Algeria	315	128	40.63 (35.2–46)	[51]
Total			11,111	2139	19 (18.5–20)	
Total Overall			11,080	2169	19.6 (18.8–20)	

**Table 3:** Prevalence of *S. aureus* in raw processed meat.

Meat Type	Year	Country	No. Tested Samples	No. *S. aureus* Positive	Prevalence % (CI)	Reference
Beef	2013	Nigeria	147	15	10,2 (5.8–16)	[33]
	2018	Egypt	100	16	20 (9.4–25)	[30]
Total			247	31	13 (8.7–17)	

**Table 4:** Prevalence of *S. aureus* in ready-to-eat meat.

Meat Type	Year	Country	No. Tested Samples	No. *S. aureus* Positive	Prevalence % (CI)	Reference
Chicken	2015–2016	South Africa	45	3.75	8.3 (2.5–21)	[54]

**Table 5:** Characteristics of antimicrobial resistance of *S. aureus* from meat and meat products in African countries.

Antimicrobial Classes	Antimicrobial Agents	Number Tested	Number Resistant	Percentage % (CI)
Penicillins Amino-penicillins Penicillin-resistant penicillin	Penicillin G	1981	363	18 (16.5–20)
	Ampicillin	229	114	50 (43.1–56)
	Oxacillin	2071	259	14 (11.1–14)
Cephalosporins	Ceftaroline	126	29	23 (15.7–33)
	Cefoxitin	1919	164	9 (7.3–10)
Glycopeptides	Vancomycin	1951	14	0.7 (0.4–1)
Aminoglycosides	Gentamicin	2085	20	1 (0.1–1)
	Amikacin	96	19	19.79 (12.4–29)
	Kanamycin	1881	97	5 (4.2–6)
Macrolides	Erythromycin	2049	222	10.8 (9.5–12)
Tetracyclines	Tetracycline	1865	123	6.5 (5.5–8)
	Doxycycline	136	37	27 (19.9–37)
	Minocycline	101	12	11 (6.3–20)
Fluoroquinolones	Ciprofloxacin	248	7	2.8 (1.1–6)
	Ofloxacin	54	31	57 (43.2–71)
Lincosamides	Clindamycin	189	64	33.8 (27.2–64)
Folate pathway inhibitors	Trimethoprim/sulphonamides	1949	51	2.6 (2–3)
Phenicols	Chloramphenicol	142	5	3.5 (0.9–6)
Oxazolidinones	Linezolid	102	9	8.8 (4.1–16)

References: [25,26,27,28,29,30,32,33,47,52,54,59,70,71,72,73,74,75].

**Table 6:** Antimicrobial resistance genes in *S. aureus* from different meat products in African countries.

Country	Meat Type	Type of Strain (Number Tested)	Resistance Genes Detected	Reference
Tunisia	Chicken	MSSA (22)	*erm(A)*, *erm(C)*, *tet(M)*, *tet(K)*, *erm(T)*, *tet(L)*, *aph (3′)-IIIa*, *ant (4′)*, *msrA*	[47]
South Africa	Beef, sheep, pork	MSSA (98)	*blaZ*, *tet(K)*, *tet(M)*, *msrA*, *ant (4′)-la*, *aph (3′)-1-IIIa*	[26]

**Table 7:** Sequence type of meat isolates from Tunisia.

Species	Type of Strain (Number)	*Spa* Type	Sequence Type (ST)	Origin of the Sample	Reference
Chicken	MSSA (2)	t899	ST 398	Poultry market meat	[47]
Chicken	MSSA (1)	t13938	ST 398	Supermarket meat	[47]
Veal	MSSA (1)	t034	ST 398	Butchery meat	[47]
Chicken	MSSA (1)	t005	ST 22	Poultry market meat	[47]
Sheep	MSSA (1)	t008	ST 8	Butchery meat	[47]

**Table 8:** Prevalence of methicillin-resistant *S. aureus* (MRSA) in different types of meat from Africa.

Meat Type	Year	Country	Studied Samples	MRSA Positive	MRSA Detection Method	Prevalence % (CI)	Reference
Raw beef meat	2015	SA	176	30	PCR ^1^	17 (11.8–23)	[92]
	2016	Nigeria	40	10	PCR	25 (12.7–41)	[46]
	2016	SA & Cameron	144	5	WGS ^2^	3.47 (1.1–8)	[70]
	2018	Egypt	100	4	PCR	4 (1.1–10)	[30]
	2018	SA	500	1	PCR	0.2 (0–1)	[26]
Total			816	50		6.12 (4.6–8)	
Raw pork meat	2015	Nigeria	200	18	Oxacillin disc-Kirby Bauer disk diffusion	9 (5.4–14)	[93]
	2015	SA	176	20	PCR	11.36 (7.1–17)	[92]
	2016	Nigeria	56	14	PCR	25 (14.4–38)	[46]
Total			432	52		12 (9.1–15)	
Raw poultry	2010–2011	Tunesia	43	2	PCR	4.65 (0.6–16)	[47]
	2013	Egypt	50	24	Latex-slide agglutination	48 (33.7–63)	[73]
	2016	Nigeria	30	6	PCR	20 (7.7–39)	[46]
	2017	SA	194	92	PCR	47 (40.2–55)	[72]
	2017	Nigeria	1800	15	PCR	0.8 (0.5–1)	[53]
	2018	Egypt	81	12	PCR	14.8 (7.9–24)	[75]
	2018	Egypt	80	8	PCR	10 (4.4–19)	[71,74]
	2019	SA	145	5	Real-time PCR	3.4 (1.1–8)	[71]
Total			2423	164		6.76 (5.8–8)	
Processed poultry	2013	Egypt	75	27	Latex-slide agglutination	36 (25.2–48)	[73]
Total			3746	293		7.8 (7–9)	

^1^ Polymerase chain reaction; ^2^ Whole-genome sequencing.

**Table 9:** Characteristics of antimicrobial resistance of methicillin-resistant *S. aureus* (MRSA) from meat and meat products in African countries.

Antimicrobial Classes	Antimicrobial Agents	Number of Tested	Number of Resistant	Percentage % (CI)
Penicillins Amino-penicillins Penicillin-resistant penicillin	Penicillin G	49	46	93 (83.1–99)
	Ampicillin	127	81	63 (54.8–72)
	Amoxicillin	52	47	90 (79–97)
	Oxacillin	80	76	95 (87.7–99)
	Methicillin	28	28	100 (87.7–100)
	Flucloxacillin	18	16	89 (65.3–99)
Cephalosporins	Cephalexin	18	17	94 (72.7–100)
	Cefoxitin	131	99	75 (67.3–83)
	Cefotaxime	4	4	100 (39.8–100)
	Cefuroxime	15	13	86 (59.5–98)
Glycopeptides	Vancomycin	92	25	27 (18.4–37)
Aminoglycosides	Gentamicin	114	54	47 (37.9–57)
	Amikacin	6	3	50 (11.8–88)
	Kanamycin	16	11	68 (41.3–88)
	Streptomycin	96	30	31 (22.2–42)
Macrolides	Erythromycin	150	94	62 (54.4–70)
Tetracyclines	Tetracycline	134	123	91 (85.8–96)
	Doxycycline	16	11	68 (41.3–89)
	Oxytetracycline	62	50	80 (68.6–90)
Fluoroquinolones	Ciprofloxacin	17	14	82 (56.6–96)
	Enrofloxacin	12	7	58 (27.7–85)
	Nalidixic acid	54	52	96 (87.3–100)
Lincosamides	Clindamycin	39	37	95 (82.7–99)
Folate pathway inhibitors	Trimethoprim/sulphonamides	146	63	43 (35–52)
Phenicols	Chloramphenicol	35	25	71 (53.7–85)

References: [26,30,46,47,53,70,71,72,73,74,75,92,93].

**Table 10:** Antimicrobial resistance genes of MRSA from African countries.

Country	Meat Type	Typed Strain (Number Tested)	Resistance Genes Detected	Reference
South Africa	Beef	MRSA (1)	*mecA*	[26]
Nigeria	Beef, chicken, pork	MRSA (49)	*mecA*	[46]
Tunisia	Chicken	MRSA (2)	*mecA*, *ermC*, *tetM*	[47]
South Africa	Chicken carcass	MRSA (2)	*mecA*, *blaZ*, *aac(6′)-aph(″)*, *erm(C)*, *mph*	[71]
Egypt	Camel carcass	MR (25)-VRSA (14)	*mecA, vanA, vanB*	[32]
Egypt	Beef meat	MRSA (4)	*mecA*, *tetK*, *vanA*	[30]
South Africa	Broiler chicken	MRSA (73)	*mecA*, *blaZ*, *tetK*	[72]

**Table 11:** Sequence tMype and *spa* type of different meat isolates from two African countries.

Species	Type of Strain (Number)	*Spa* Type	Sequence Type (ST)	Origin of the Sample	Reference
Chicken	MRSA	t102	ST30	Farm	[47]
Chicken	MRSA	t4358	ST398	Poultry market	[47]
Carcass	MRSA	t1257	ST612	Retail point	[71]
Chicken	MRSA	t037	ST239	Slaughterhouse	[110]

## Data Availability

Not applicable.
